# Variation Across Hospitals in In-Hospital Cardiac Arrest Incidence Among Medicare Beneficiaries

**DOI:** 10.1001/jamanetworkopen.2021.48485

**Published:** 2022-02-28

**Authors:** Tyler P. Rasmussen, Danielle J. Riley, Mary Vaughan Sarazin, Paul S. Chan, Saket Girotra

**Affiliations:** 1 Division of Cardiovascular Medicine, Department of Internal Medicine, University of Iowa, Iowa City; 2 Department of Epidemiology, University of Iowa, Iowa City; 3 Center for Access and Delivery Research and Evaluation, Veterans Affairs Medical Center, Iowa City; 4 Mid-America Heart Institute, University of Missouri, Kansas City

## Abstract

**Importance:**

Although survival for in-hospital cardiac arrest (IHCA) has improved substantially over the last 2 decades, survival rates have plateaued in recent years. A better understanding of hospital differences in IHCA incidence may provide important insights regarding best practices for prevention of IHCA.

**Objective:**

To determine the incidence of IHCA among Medicare beneficiaries, and evaluate hospital variation in incidence of IHCA.

**Design, Setting, and Participants:**

This observational cohort study analyzes 2014 to 2017 data from 170 hospitals participating in the Get With The Guidelines-Resuscitation registry, linked to Medicare files. Participants were adults aged 65 years and older. Statistical analysis was performed from January to December 2021.

**Exposures:**

Case-mix index, teaching status, and nurse-staffing.

**Main Outcomes and Measures:**

Hospital incidence of IHCA among Medicare beneficiaries was estimated as the number of IHCA patients divided by the total number of hospital admissions. Multivariable hierarchical regression models were used to calculate hospital incidence rates adjusted for differences in patient case-mix and evaluate the association of hospital variables with IHCA incidence.

**Results:**

Among a total of 4.5 million admissions at 170 hospitals, 38 630 patients experienced an IHCA during 2014 to 2017. Among the 38 630 patients with IHCAs, 7571 (19.6%) were non-Hispanic Black, 26 715 (69.2%) were non-Hispanic White, and 16 732 (43.3%) were female; the mean (SD) age at admission was 76.3 (7.8) years. The median risk-adjusted IHCA incidence was 8.5 per 1000 admissions (95% CI, 8.2-9.0 per 1000 admissions). After adjusting for differences in case-mix index, IHCA incidence varied markedly across hospitals ranging from 2.4 per 1000 admissions to 25.5 per 1000 admissions (IQR, 6.6-11.4; median odds ratio, 1.51 [95% CI, 1.44-1.58]). Among hospital variables, a higher case-mix index, higher nurse staffing, and teaching status were associated with a lower hospital incidence of IHCA.

**Conclusions and Relevance:**

This cohort study found that the incidence of IHCA varies markedly across hospitals, and hospitals with higher nurse staffing and teaching status had lower IHCA incidence rates. Future studies are needed to better understand processes of care at hospitals with exceptionally low IHCA incidence to identify best practices for cardiac arrest prevention.

## Key Points

**Question:**

What is the incidence of in-hospital cardiac arrest (IHCA) among older patients and does the incidence vary across hospitals?

**Findings:**

In an observational cohort study using Get With the Guidelines-Resuscitation data linked with Medicare, the overall IHCA incidence was 8.53 per 1000 admissions with large variation in incidence across hospitals (range, 2.4-25.5), even after adjusting for differences in case-mix severity. Higher nurse staffing and teaching status was associated with a lower hospital incidence of IHCA.

**Meaning:**

These findings suggest that incidence of IHCA varies markedly across US hospitals, even after adjusting for patient case-mix.

## Teaser

This cohort study examines the variation across hospitals in incidence of in-hospital cardiac arrest among Medicare beneficiaries.

## Introduction

In-hospital cardiac arrest (IHCA) affects nearly 290 000 hospitalized patients each year in the United States^1^ and is associated with poor survival and a high risk of neurological disability among survivors.^2,3^ Over the past decade, hospitals have devoted considerable effort toward improving IHCA survival by improving care delivery during the acute resuscitation phase^4,5,6^ and the postresuscitation phase.^7^ Despite these efforts, IHCA survival rates have plateaued in recent years, with mean survival of approximately 25%.^2,8,9^ Given the high incidence and low survival for IHCA, efforts targeted toward prevention of IHCA could be impactful.

Cardiac arrest in hospitalized patients is rarely sudden, and may be a result of inappropriate triage, delays in diagnosis, or treatment in deteriorating patients. Prior studies have found that nearly 40% of patients who experience IHCA on hospital wards have abnormal vital signs during the preceding 1 to 4 hours, which often go unrecognized.^10,11,12^ It is possible that some hospitals have developed innovations or systems of care (eg, rapid response teams) that identify and treat unstable patients before they progress to IHCA. Understanding the extent to which the incidence of IHCA varies across hospitals in the US and the factors associated with this variation is a critical first step toward identifying best practices for IHCA prevention.

To address this gap in knowledge, we linked Get With the Guidelines–Resuscitation (GWTG-R) database with Medicare inpatient claims summarized by hospital to calculate hospital rates of IHCA incidence among Medicare beneficiaries and identify hospital factors associated with IHCA incidence. An improved understanding of variation in IHCA incidence and the associated factors is important for developing strategies for reducing the burden of IHCA in the United States.

## Methods

The University of Iowa institutional review board deemed this cohort study exempt and granted a waiver of informed consent because this study used deidentified data. We followed the Strengthening the Reporting of Observational Studies in Epidemiology (STROBE) reporting guideline.

### Data Sources

This study was conducted using the following data sources for the study period of 2014 to 2017: (1) GWTG-R, a large, prospective, multisite registry of IHCA events in the United States that includes detailed information on all confirmed IHCA cases submitted by participating hospitals using standardized Utstein-style definitions,^13^ (2) Center for Medicare and Medicaid (CMS) Part A data that include hospitalization data on all Medicare beneficiaries, (3) annual case-mix index files for each hospital, which are publicly reported by CMS and include a hospital measure of case-mix severity, and (4) the American Hospital Association data for information on hospital variables.

### Data Linkage

First, using Medicare Part A data, we identified all patients aged 65 years and older admitted to an acute care hospital in the US during 2014 to 2017. We chose this time frame as we had access to Medicare data through 2017. For each hospital, we summed the total number of hospitalizations during each year. Next, we merged this data set with the case-mix index data for that year, using the CMS hospital identifier available in both data sets. The case-mix index was based on the relative weight assigned to the diagnosis-related groups of all hospital discharges at each hospital and is a measure of illness severity of admitted patients. Finally, the aforementioned hospital-level data set was merged with hospital-level GWTG-R data and American Hospital Association data. This last step was performed by the Biostatistics Core at University of Pennsylvania (data analytic center for GWTG-R) using a master file that included a crosswalk between the hospital identifiers in the CMS, American Hospital Association, and the GWTG-R data. Upon completion of this linkage, a deidentified data set was provided to the study team for analysis to ensure that the study authors remained blinded to the identity of the hospitals included in the study.

### Study Cohort

**Figure 1.**
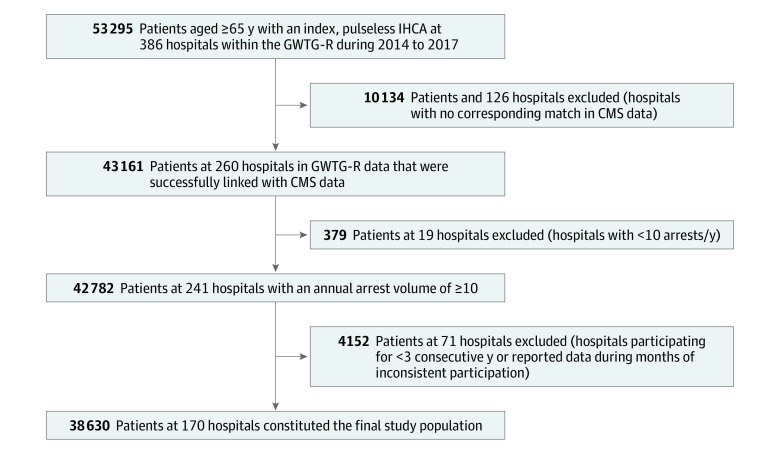
Study Cohort Flowchart CMS indicates Centers for Medicare & Medicaid Services; GWTG-R, Get With the Guidelines-Resuscitation; and IHCA, in-hospital cardiac arrest.

Within patient-level GWTG-R data, we identified 386 hospitals that included 53 295 patients aged 65 years and older with an index, pulseless cardiac arrest in an inpatient location during 2014 to 2017 (Figure 1). Of these 386 hospitals, 260 (67.3%) hospitals (43 161 patients) had a corresponding match in the linked data set, which provided total number of hospitalizations and case-mix index from CMS data. To ensure that the IHCA incidence rates were stable, we excluded 19 hospitals that reported fewer than 10 IHCA events per year, and 71 hospitals that did not participate in GWTG-R for at least 3 consecutive years. Our final study sample consisted of 38 630 adult patients at 170 hospitals representing 4 473 897 admissions of Medicare enrollees.

### Study Variables and Outcomes

The primary outcome of interest was hospital incidence rate of IHCA among Medicare beneficiaries, adjusted for case-mix index. The annual incidence of IHCA for each hospital was calculated by dividing the total number of IHCA events in patients aged 65 years or greater (obtained from GWTG-R) by the total number of hospital admissions during the same year (obtained from CMS data). For hospitals that did not participate in GWTG-R for the entire 4-year study period, we prorated the total number of admissions (denominator) by the number of months of participation and divided by number of years of participation in GWTG-R to calculate their overall annual incidence of IHCA.^14^

Hospital-level variables included bed size, geographic census region, rurality, ownership type, teaching status, and trauma center designation from the American Hospital Association data. We also calculated a measure of nurse staffing at each hospital as ratio of the number of full-time nurse equivalents and the total inpatient days available in the American Hospital Association data.^14,15^ Patient-level demographic and clinical characteristics for IHCA patients were identified from the GWTG-R. Self-reported demographic characteristics included age at hospital admission, gender, and race and ethnicity. Clinical characteristics included presence of comorbid conditions including current or prior heart failure, current or prior myocardial infarction, diabetes, central nervous system depression, acute neurological nonstroke event, acute stroke, hepatic insufficiency, renal insufficiency, respiratory insufficiency, septicemia, pneumonia, hypotension, major trauma, malignancy, and metabolic or electrolyte abnormality. We also collected year of admission, first pulseless rhythm, event location, time of cardiac arrest (weekday, weeknight, or weekend), the use of a hospital-wide cardiopulmonary arrest alert, and interventions in place at time of arrest (ventilatory support, invasive airway, vasoactive medications, and dialysis).

### Statistical Analysis

We used descriptive statistics for summarizing patient and hospital characteristics—mean (SD) or median (IQR) for continuous variables, and number (percentage) for categorical variables. Categorical variables were compared using χ^2^ or Fisher exact test. Trend tests were calculated using Cochran-Armitage or Cochran-Mantel-Haenzei tests, where appropriate. *P* < .05 was considered statistically significant. Next, we constructed a hierarchical random effects regression model to calculate case-mix adjusted rates of IHCA incidence for each hospital. Hierarchical models account for clustering of patients within hospitals thus reducing overestimation of statistical significance.^16^ In these models, IHCA events per-hospital were modeled using a logit link and binomial distribution, with hospital site included as a random effect. To ensure that hospital rates of IHCA incidence were not confounded by differences in case-mix severity across hospitals, we adjusted hospital IHCA incidence rates for case-mix index obtained from CMS. Using regression coefficients from this model, we estimated each hospital’s incidence rate for IHCA as the ratio of predicted-to-expected incidence multiplied by the overall unadjusted incidence rate during the study period. Compared with the observed-to-expected ratio, the predicted-to-expected ratio does not unfairly penalize small volume hospitals by accounting for the lower precision in survival estimates from such volume hospitals.^17^ Confidence intervals for risk-adjusted incidence rates were estimated using a bootstrap approach. Specifically, 1000 hospital samples were generated with replacement, which were then used to estimate the 2.5th and 97.5th percentile risk-adjusted incidence rates over repeated modeling. To quantify the magnitude of hospital-level variation in IHCA incidence, we calculated a median odds ratio from the aforementioned hierarchical model using the variance estimate of the random hospital intercept.^18^

We categorized study hospitals into quartiles based on the case-mix adjusted IHCA incidence and examined patient- and hospital-variables across quartiles of IHCA incidence using linear regression for continuous variables and χ^2^ test for categorical variables. Finally, we evaluated the association between hospital variables and case-mix adjusted IHCA incidence using the same hierarchical regression models previously described. All analyses were performed using SAS software version 9.4 (SAS Institute) from January to December 2021.

## Results

**Figure 2.**
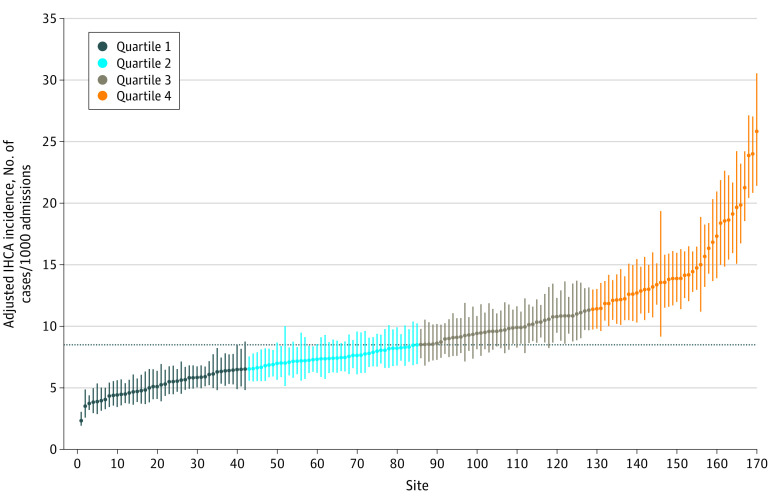
Hospital Variation in the Case-Mix Adjusted Incidence of IHCA Among Medicare Beneficiaries Per 1000 Admission The x-axis represents each hospital arranged in the ascending order of IHCA incidence, and the y-axis represents the IHCA incidence expressed as per-1000 admissions. Error bars represent 95% CIs. The horizontal dotted line represents the median adjusted IHCA incidence (8.5) for our study. The graph is color coded for dark blue = quartile 1, cyan = quartile 2, brown = quartile 3, and orange = quartile 4.

In the study cohort of 170 hospitals, there were 38 630 IHCAs in patients aged at least 65 years of age among 4 473 897 Medicare admissions during 2014 to 2017. Among the 38 630 patients with IHCAs, 7571 were non-Hispanic Black, 26 715 were non-Hispanic White, and 16 732 were female; the mean (SD) age at admission was 76.3 (7.8) years. The median annualized hospital incidence rate of IHCA among Medicare patients was 8.5 per 1000 admissions (95% CI, 8.2-9.0 per 1000 admissions). After adjustment for case-mix, hospital incidence of IHCA varied by more than 10-fold from 2.4 to 25.5 per 1000 admissions (IQR, 6.5 to 11.4 per 1000 admissions) (Figure 2). Study hospitals were categorized into quartiles based on the incidence of IHCA (Q1: <6.5 IHCAs per 1000 admissions; Q2: 6.5-8.5 per 1000 admissions; Q3: 8.6-11.4 per 1000 admissions; and Q4: >11.4 per 1000 admissions).

Table 1 includes hospital characteristics overall, and and when stratified by hospital quartiles of IHCA incidence. Study hospitals were evenly distributed across geographic census regions, and a majority of them were located in urban areas (152 hospitals [89.4%]) , were nonprofit (125 hospitals [73.6%]) and were either major teaching centers (42 hospitals [24.7%]) or minor teaching centers (99 hospitals [58.2%]). There were 43 large hospitals (25.3%) with at least 500 beds. Compared with hospitals in the lowest quartile of IHCA incidence (Q1), hospitals in the highest quartile (Q4) were more likely to be in the South Atlantic (33.4% vs 11.4%) or South-Central region (31.1% vs 6.8%) and more likely to have private ownership (17.8% vs 2.3%). The mean nurse staffing was 9.0 nursing full-time equivalents (FTEs) per 1000 patient days overall. Hospitals in the lowest quartile of IHCA incidence had higher levels of nurse staffing compared with hospitals in the highest quartile of IHCA incidence (9.6 vs 8.2).

**Table 1.** Hospital Characteristics by Quartiles of Case-Mix Adjusted IHCA Incidence^a^

Hospital characteristics	Hospitals, No. (%)					*P* value for trend
	Total (n = 170)	Quartiles of case-mix adjusted IHCA incidence rates				
		Q1 < 6.5 (n = 44)	Q2 6.5-8.5 (n = 41)	Q3 8.6-11.4 (n = 40)	Q4 ≥ 11.5 (n = 45)	
Case-mix index						
Median (IQR)	1.7 (1.6-1.8)	1.6 (1.5-1.8)	1.7 (1.6-1.8)	1.7 (1.6-1.8)	1.7 (1.6-1.8)	.13
Mean (SD)	1.7 (0.2)	1.6 (0.3)	1.7 (0.2)	1.7 (0.2)	1.7 (0.3)	
Nurse FTEs/1000 patient days						
Median (IQR)	9.0 (7.6-10.2)	9.6 (8.5-10.8)	8.6 (7.4-9.9)	9.0 (7.9-10.6)	8.2 (6.7-10.0)	.02
Mean (SD)	9.3 (2.4)	10.0 (2.5)	9.2 (2.6)	9.2 (1.9)	8.6 (2.4)	
US Census region						
North Mid-Atlantic	33 (19.4)	14 (31.8)	6 (14.6)	9 (22.5)	4 (8.9)	.008
South Atlantic	36 (21.2)	5 (11.4)	6 (14.6)	10 (25.0)	15 (33.4)	
North Central	38 (22.4)	11 (25.0)	13 (31.8)	8 (20.0)	6 (13.3)	
South Central	28 (16.5)	3 (6.8)	6 (14.6)	5 (12.5)	14 (31.1)	
Mountain Pacific	32 (18.8)	11 (25.0)	9 (22.0)	6 (15.0)	6 (13.3)	
Unknown	3 (1.7)	0	1 (2.4)	2 (5.0)	0 (0.0)	
Facility location						
Rural	10 (5.9)	3 (6.8)	0 (0.0)	2 (5.0)	5 (11.1)	.43
Urban	152 (89.4)	40 (90.9)	38 (92.7)	36 (90.0)	38 (84.5)	
Unknown	8 (4.7)	1 (2.3)	3 (7.3)	2 (5.0)	2 (4.4)	
Ownership						
Nonprofit	125 (73.6)	39 (88.6)	32 (78.1)	28 (70.0)	26 (57.8)	.02
Public	24 (14.1)	3 (6.8)	8 (19.5)	4 (10.0)	9 (20.0)	
Private	14 (8.2)	1 (2.3)	0	5 (12.5)	8 (17.8)	
Unknown	7 (4.1)	1 (2.3)	1 (2.4)	3 (7.5)	2 (4.4)	
Academic teaching status						.36
Major teaching	42 (24.7)	10 (22.7)	7 (17.1)	13 (32.5)	12 (26.7)	.36
Minor teaching	99 (58.2)	27 (61.4)	28 (68.3)	21 (52.5)	23 (51.1)	
Nonteaching	26 (15.3)	7 (15.9)	5 (12.2)	4 (10.0)	10 (22.2)	
Unknown	3 (1.8)	0 (0.0)	1 (2.4)	2 (5.0)	0 (0.0)	
Trauma center						
Yes	117 (68.8)	33 (75.0)	28 (68.3)	29 (72.5)	27 (60.0)	.73
No	32 (18.8)	8 (18.2)	8 (19.5)	6 (15.0)	10 (22.2)	
Unknown	21 (12.4)	3 (6.8)	5 (12.2)	5 (12.5)	8 (17.8)	
Hospital bed size, No.						
<200	27 (15.9)	12 (27.3)	7 (17.1)	2 (5.0)	6 (13.3)	.10
200-499	94 (55.3)	25 (56.8)	18 (43.9)	26 (65.0)	25 (55.6)	
≥500	43 (25.3)	7 (15.9)	13 (31.7)	10 (25.0)	13 (28.9)	
Unknown	6 (3.5)	0	3 (7.3)	2 (5.0)	1 (2.2)	

Abbreviations: FTEs, full-time equivalents; IHCA, in-hospital cardiac arrest.

^a^ Table shows the association between hospital-level characteristics and case-mix adjusted IHCA incidence divided into quartiles. Categorical variables were compared using χ^2^ or Fisher exact test. Trend tests were calculated using Cochran-Armitage or Cochran-Mantel-Haenzei tests, where appropriate.

Table 2 shows characteristics of IHCA patients stratified by quartiles of hospital IHCA incidence. Compared with Q1 hospitals, patients who experienced an IHCA at Q4 hospitals were younger (median [IQR] age: 75 [70-82] years vs 76 [70-83] years; *P* < .001), more likely to be non-Hispanic Black (4102 patients [30.3%] vs 522 patients [8.4%]; *P* < .001), and female (6060 patients [44.8%] vs 2588 patients [41.7%]; *P* < .001), and had a higher overall burden of preexisting conditions (eg, current heart failure: 2280 (16.9%) vs 792 (12.8%); *P* < .001). Patients in Q4 hospitals were also more likely to have an initial rhythm of asystole or pulseless electrical activity (10 750 patients [79.4%] vs 4689 patients [75.6%]; *P* < .001), cardiac arrest in an ICU location (6341 patients [46.9%] vs 2686 patients [43.3%]; *P* < .001), and receive mechanical ventilation at the time of cardiac arrest (5595 patients [41.3%] vs 2222 patients [35.8%]; *P* < .001) (Table 2).

**Table 2.** Patient Characteristics by Quartiles of Case-Mix Adjusted IHCA Incidence^a^

Patient characteristics	Patients, No. (%)					*P* value for trend
	Total (n = 38 630)	Quartiles of case-mix adjusted IHCA incidence rates				
		Q1 < 6.5 (n = 6199)	Q2 6.5-8.5 (n = 8094)	Q3 8.6-11.4 (n = 10 804)	Q4 ≥ 11.5 (n = 13 533)	
Age at admission, years						
Median (IQR)	75 (70-82)	76 (70-83)	75 (70-82)	75 (70-82)	75 (70-82)	<.001
Mean (SD)	76.3 (7.8)	76.7 (7.9)	76.1 (7.7)	76.4 (7.8)	76.0 (7.7)	
Gender						
Male	21 898 (56.7)	3611 (58.3)	4635 (57.3)	6179 (57.2)	7473 (55.2)	<.001
Female	16 732 (43.3)	2588 (41.7)	3459 (42.7)	4626 (42.8)	6060 (44.8)	
Race and ethnicity						
Non-Hispanic Black	7571 (19.6)	522 (8.4)	1026 (12.7)	1921 (17.8)	4102 (30.3)	<.001
Non-Hispanic White	26 715 (69.2)	4830 (77.9)	6171 (76.3)	7671 (71.0)	8043 (59.4)	
Other	2291 (5.9)	391 (6.3)	400 (4.9)	771 (7.1)	729 (5.4)	
Unknown	2053 (5.3)	456 (7.4)	497 (6.1)	441 (4.1)	659 (4.9)	
Preexisting conditions						
Current heart failure	6479 (16.8)	792 (12.8)	1215 (15.0)	2192 (20.3)	2280 (16.9)	<.001
Prior heart failure	10 397 (26.9)	1727 (27.9)	2014 (24.9)	2952 (27.3)	3704 (27.4)	.25
Current MI	5883 (15.2)	869 (14.0)	1195 (14.8)	1776 (16.4)	2043 (15.1)	.03
Prior MI	6296 (16.3)	1116 (18.0)	1138 (14.1)	1900 (17.6)	2142 (15.8)	.23
Diabetes	14147 (36.6)	2221 (35.8)	2785 (34.4)	3882 (35.9)	5259 (38.9)	<.001
Baseline depression in CNS function	3125 (8.1)	512 (6.3)	441 (5.5)	860 (8.0)	1312 (9.7)	<.001
Acute CNS nonstroke event	2515 (6.5)	339 (5.5)	403 (5.0)	827 (7.7)	946 (7.0)	<.001
Acute stroke	1739 (4.5)	275 (4.4)	298 (3.7)	493 (4.6)	673 (5.0)	.002
Hepatic insufficiency	1921 (5.0)	302 (4.9)	314 (3.9)	522 (4.8)	783 (5.8)	<.001
Renal insufficiency	14 498 (37.5)	2276 (36.7)	2978 (36.8)	4176 (38.7)	5068 (37.5)	.15
Respiratory insufficiency	17 763 (46.0)	2407 (38.8)	3387 (41.9)	5370 (49.7)	6599 (48.8)	<.001
Septicemia	6758 (17.5)	972 (15.7)	1342 (16.6)	2012 (18.6)	2432 (18.0)	<.001
Pneumonia	5736 (14.9)	830 (13.4)	1110 (13.7)	1650 (15.3)	2146 (15.9)	<.001
Hypotension or hypoperfusion	10 053 (26.0)	1399 (22.6)	1697 (21.0)	2732 (25.3)	4225 (31.2)	<.001
Major trauma	1202 (3.1)	138 (2.2)	229 (2.8)	380 (3.5)	455 (3.4)	<.001
Metastatic or hematologic cancer	4421 (11.4)	733 (11.8)	808 (10.0)	1244 (11.5)	1636 (12.1)	.02
Metabolic or electrolyte abnormality	8196 (21.2)	1196 (19.3)	1245 (15.4)	2787 (25.8)	2968 (21.9)	<.001
Year of admission						
2014	9058 (23.4)	1335 (21.5)	1705 (21.1)	2714 (25.1)	3304 (24.4)	<.001
2015	9892 (25.6)	1574 (25.4)	2065 (25.5)	2712 (25.1)	3541 (26.2)	
2016	9871 (25.6)	1656 (26.7)	2123 (26.2)	2863 (26.5)	3329 (23.8)	
2017	9809 (25.4)	1634 (26.4)	2201 (27.2)	2515 (23.3)	3459 (25.6)	
First pulseless rhythm						
Asystole/PEA	29 891 (77.4)	4689 (75.6)	6017 (74.3)	8435 (78.1)	10 750 (79.4)	<.001
PVT/VF	5951 (15.4)	1078 (17.4)	1293 (16.0)	1631 (15.1)	1949 (14.4)	
Unknown	2788 (7.2)	432 (7.0)	784 (9.7)	738 (6.8)	834 (6.2)	
Event location						
Intensive care unit	17 700 (45.8)	2686 (43.3)	3724 (46.0)	4949 (45.8)	6341 (46.9)	<.001
Procedural	3386 (8.8)	602 (9.7)	612 (7.6)	939 (8.7)	1233 (9.1)	
Emergency department	3290 (8.5)	401 (6.5)	505 (6.2)	987 (9.1)	1397 (10.3)	
Ward	6806 (17.6)	1217 (19.6)	1666 (20.6)	2069 (19.2)	1854 (13.7)	
Telemetry	7098 (18.4)	1219 (19.7)	1506 (18.6)	1779 (16.5)	2594 (19.2)	
Other	350 (0.9)	74 (1.2)	81 (1.0)	81 (0.7)	114 (0.8)	
Time of arrest						
Weekday (8AM-5PM)	12 921 (33.5)	2139 (34.5)	2634 (32.5)	3588 (33.2)	4560 (33.7)	<.001
Weeknight (5PM-8AM)	15 395 (39.9)	2448 (39.5)	3283 (40.6)	4300 (39.8)	5364 (39.6)	
Weekend	10 187 (26.4)	1592 (25.7)	2166 (26.8)	2846 (26.3)	3583 (26.5)	
Unknown	127 (0.2)	20 (0.3)	11 (0.1)	70 (0.7)	26 (0.2)	
Activated hospital-wide cardiopulmonary arrest alert						
Yes	29 321 (75.9)	5045 (81.4)	6591 (81.4)	7651 (70.8)	10 034 (74.1)	<.001
No	9309 (24.1)	1154 (18.6)	1503 (18.6)	3153 (29.2)	3499 (25.9)	
Interventions in place						
Assisted or mechanical ventilation	15 143 (39.2)	2222 (35.8)	2828 (34.9)	4498 (41.6)	5595 (41.3)	<.001
Invasive airway	8509 (22.0)	1137 (18.3)	1482 (18.3)	2606 (24.1)	3284 (24.3)	<.001
IV or IO vasoactive agents	8532 (22.1)	1418 (22.9)	1580 (19.5)	2411 (22.3)	3123 (23.1)	.006
Dialysis or extracorporeal filtration	941 (2.4)	87 (1.4)	273 (3.4)	219 (2.0)	362 (2.7)	.01

Abbreviations: CNS, central nervous system; IHCA, in-hospital cardiac arrest; IV, intravenous; IO, intraosseus; MI, myocardial infarction; PEA, pulseless electrical activity; PVT, polymorphic ventricular tachycardia; VF, ventricular fibrillation.

^a^ Table shows the association between patient-level characteristics and case-mix adjusted IHCA incidence divided into quartiles. Categorical variables were compared using χ^2^ or Fisher exact test. Trend tests were calculated using Cochran-Armitage or Cochran-Mantel-Haenzei tests, where appropriate.

After adjusting for case-mix index, the median odds ratio for hospital IHCA incidence was 1.51 (95% CI, 1.44-1.58), suggesting that the relative odds that a hospitalized patient at one randomly selected hospital would experience an IHCA were 51% higher compared with a similar patient admitted at another randomly selected hospital with identical case-mix. In addition to case-mix index, higher nurse staffing was associated with a lower incidence of IHCA (odds ratio [OR], 0.96 [95% CI, 0.92-0.99]; *P* = .007) (Table 3). Hospital teaching status was also associated with IHCA incidence, although the effect estimate was similar for minor teaching and nonteaching hospitals, an association was found only for minor teaching hospitals (OR, 0.74 [95% CI, 0.58-0.93]) (Table 3). The addition of hospital variables to the hierarchical model explained 12% of hospital variation in IHCA, and the median OR after adjusting for hospital variables was 1.47 (95% CI, 1.41-1.54).

**Table 3.** Association of Hospital Variables With Incidence of IHCA

Hospital characteristics (N = 136)	Adjusted	
	OR (95% CI)^a^	*P* value
Case-mix index	0.73 (0.71-0.74)	<.001
Nurse FTEs/1000 patient days	0.96 (0.92-0.99)	.007
Region		
North Mid-Atlantic	1 [Reference]	.25
South Atlantic	1.22 (0.96-1.55)	
North Central	1.19 (0.94-1.50)	
South Central	1.34 (1.03-1.74)	
Mountain Pacific	1.23 (0.97-1.56)	
Facility location		
Rural	1.10 (0.81-1.50)	.54
Urban	1 [Reference]	
Ownership		
Nonprofit	1 [Reference]	.17
Public	1.22 (0.98-1.52)	
Private	1.28 (0.90-1.81)	
Unknown	0.92 (0.59-1.43)	
Academic teaching status		
Major	1 [Reference]	.03
Minor	0.74 (0.58-0.93)	
None	0.78 (0.57-1.07)	
Trauma center		
Yes	1 [Reference]	.59
No	1.05 (0.87-1.27)	
Hospital bed size, No.		
<200	0.78 (0.58-1.05)	.11
200-499	0.98 (0.79-1.21)	
≥500	1 [Reference]	

Abbreviations: FTEs, full-time equivalents; IHCA, in-hospital cardiac arrest; OR, odds ratio.

## Discussion

In this large cohort study of 170 GWTG-R hospitals linked with Medicare data, we found that the median risk-adjusted incidence of IHCA among Medicare beneficiaries was 8.5 per 1000 admissions. Importantly, we found substantial variation in the incidence of IHCA across study hospitals, ranging from 2.4 per-1000 to 25.5 per-1000 admissions with a median odds ratio of 1.51. In adjusted analyses, we found that higher levels of nurse staffing and minor teaching status was associated with lower incidence of IHCA. Furthermore, adjustment for differences in hospital variables including case-mix explained only 12% of the variation in IHCA incidence, suggesting that unmeasured differences in hospital care processes likely account for the large variation in IHCA incidence. Our findings are important and merit further discussion.

The overall incidence of IHCA in our study (8.5 per1000 admissions) was higher than the reported incidence of 4.02 per 1000 admissions in an earlier GWTG-R study that used 2000 to 2009 data.^14^ Although recent studies have reported an increase in the incidence of IHCA,^1^ we attribute the higher incidence in the current study to the inclusion of only Medicare beneficiaries who represent an older, higher risk population. Although the incidence rate of IHCA in our study may not be generalizable to younger patients, there are several strengths of our study that are important to emphasize. First, linkage with Medicare files provided accurate data on the total number of admissions, which was the denominator used for calculation of IHCA incidence. In contrast, prior studies have obtained data on total admissions from the American Hospital Association data, which does not differentiate adult from pediatric admissions. Therefore, IHCA incidence rates are likely to be underestimated in these studies especially at hospitals that treat a large number of pediatric patients.^1,19^ Second, linkage with Medicare files also provided data on case-mix severity in the denominator population of admitted patients. Adjustment for case-mix in the calculation of IHCA incidence overcomes an important limitation of prior studies because case-mix index information is not available in the American Hospital Association data.

A notable finding of our study was the large variation in IHCA incidence across hospitals, which highlights that some hospitals are able to achieve a lower incidence of IHCA compared with others, adjusted for case-mix. Numerous studies have shown that a large proportion of IHCA events in hospitalized patients may be preventable and may occur because of inappropriate triage, delays in recognition, diagnosis and treatment of life-threating conditions, or development of new problems or complications among admitted patients (ie, failure to rescue).^10,20,21^ In adjusted analyses, we found higher nurse staffing was associated with lower incidence of IHCA, which could be due to nurse surveillance of at-risk patients and prompt activation of hospital emergency response teams for patients experiencing deteriorating conditions. These findings are consistent with prior studies that have shown higher levels of nurse staffing to be associated with lower hospital mortality and higher quality of care.^22,23^ Unlike other hospital structural variables such as teaching status, which are usually not modifiable, nurse staffing is modifiable. Future studies are needed to confirm or refute whether improving nurse staffing at hospitals with higher IHCA incidence are associated with a reduction in IHCA and other adverse patient outcomes.

We also found that adjustment for hospital variables and case-mix accounted for a small proportion of the variation in IHCA incidence across hospitals, which suggests that some hospitals may have developed innovative care processes that may enable earlier recognition and treatment of patients experiencing deteriorating conditions before they progress to IHCA. In a recent study, we found substantial differences across hospitals in the organization and structure of rapid response teams—a key hospital intervention that is focused toward preventing IHCA.^24^ Likewise, others have developed the electronic Cardiac Arrest Risk Triage system that uses real-time data in the electronic health record to forewarn bedside nurses and physicians about the risk of impending IHCA.^25,26^ Currently, neither GWTG-R nor other existing hospital databases collect information on these innovative processes of care and treatment strategies. Future research focused on hospitals that have achieved exceptionally low incidence of IHCA may yield important insights regarding care processes and organizational variables (best practices) for prevention of IHCA among hospitalized patients.^27^

### Limitations

Our study findings should be interpreted in the context of the following limitations. First, due to linkage with Medicare data, we were only able to calculate IHCA incidence among patients aged 65 years and older. Although the IHCA incidence rates may not be generalizable to younger patients, there is no reason to believe that hospitals in the highest quartile of IHCA for Medicare patients will be a lower quartile for younger patients given that processes of care for IHCA prevention do not differ for Medicare and non-Medicare patients at a hospital. Second, information on hospital-level variables was limited to only hospital structural variables, and we lacked information on modifiable hospital process for IHCA (eg, triage processes, composition, and structure of rapid response teams, quality improvement efforts, etc.) that possibly underlie variation in IHCA incidence. Based on our findings, we believe that this represents an important next area for research. Furthermore, hospital variables were missing for many hospitals, which limited analysis. Third, it is possible that the variation in IHCA incidence could be due to hospital variation with regard to do-not-resuscitation (DNR) orders, such that low incidence of IHCA at some hospitals may be due to more robust use of DNR and palliative care, as these patients would not be enrolled in the GWTG-R program. Information on DNR rates among all hospital admissions were not available in Medicare. However, to the extent that use of DNR orders represents care that is appropriate and patient-centered, this may represent high-quality care at hospitals with low IHCA incidence. Fourth, our study included linked Medicare data from 2014 to 2017, which at the time of data analysis was the most current available data for the GWTG-R registry. We have no reason to believe that these years are not representative of more contemporary data from 2018 and 2019. Finally, our study only included hospitals participating in GWTG-R and therefore our findings may not be generalizable to all hospitals. However, hospitals participating in GWTG-R likely represent facilities that have resources and commitment toward engaging in resuscitation quality improvement. Therefore, these findings may underestimate the incidence of IHCA and its variation at nonparticipating hospitals.

## Conclusions

We found that incidence of IHCA varied widely across US hospitals even after adjustment for patient case-mix index. Although higher nurse staffing levels and teaching status were associated with a lower hospital incidence of IHCA, a majority of the hospital variation in IHCA incidence remained unexplained. Further studies are warranted to better understand care delivery practices and systems of care in hospitals with low IHCA incidence to identify best practices for the prevention of IHCA.
